# Epidemiology, risk factors, clinical presentation and complications of late-onset neonatal sepsis among preterm neonates in Cyprus: a prospective case-control study

**DOI:** 10.1186/s12887-023-04359-6

**Published:** 2024-01-16

**Authors:** Paraskevi Stylianou-Riga, Theodora Boutsikou, Panayiotis Kouis, Kyriaki Michailidou, Paraskevi Kinni, Rozeta Sokou, Zoi Iliodromiti, Constantinos Pitsios, Panayiotis K. Yiallouros, Nicoletta Iacovidou

**Affiliations:** 1 Neonatal Intensive Care Unit, “Archbishop Makarios III” Hospital, Nicosia, Cyprus; 2 Respiratory Physiology Laboratory, Medical School, University of Cyprus Shakolas Educational Center of Clinical, Medicine Palaios Dromos Lefkosias Lemesou 215/6, Aglantzia, Nicosia, 2029 Cyprus; 3 Neonatal Department, Medical School, Aretaieio Hospital, National and Kapodistrian University of Athens, Athens, Greece; 4 Biostatistics Unit, Institute of Neurology and Genetics, Nicosia, Cyprus

## Abstract

**Background:**

Late-onset neonatal sepsis (LOS) is common in preterm neonates, with increasing incidence in recent years. In the present study, we examined the epidemiology, clinical presentation, and complications of LOS in Cyprus and quantified possible risk factors for the development of this condition.

**Methods:**

The study subjects were preterm neonates admitted in the Neonatal Intensive Care Unit (NICU) of Archbishop Makarios III Hospital, the only neonatal tertiary centre in Cyprus. A prospective, case-control study was designed, and carried out between April 2017-October 2018. Depending on blood culture results, preterm neonates were classified as “Confirmed LOS”: positive blood culture - microorganism isolated and LOS symptoms, “Unconfirmed LOS”: negative blood culture and LOS symptoms, and “Controls” group: negative blood culture and absence of LOS symptoms. Comparisons between the 3 groups were performed and the associations between demographic, clinical and treatment characteristics with the likelihood of LOS were assessed using univariate and multivariate logistic regression.

**Results:**

A total of 350 preterm neonates were included in the study and the incidence of LOS was 41.1%. 79 (22.6%) and 65 (18.6%) neonates were classified as “Confirmed LOS”, and “unconfirmed LOS” cases respectively while 206 (58.9%) served as controls. The rate of confirmed LOS ranged from 12.2% in moderate to late preterm neonates to 78.6% in extremely preterm neonates. In the multivariate model, we demonstrated an independent association between LOS and duration of hospitalization (OR: 1.06, 95%CI: 1.01–1.10), duration of ventilation (OR: 1.23, 95%CI: 1.07–1.43) and necrotising enterocolitis (OR: 3.41, 95%CI: 1.13–10.25).

**Conclusions:**

The present study highlights the epidemiology of LOS in preterm neonates in Cyprus and its association with the duration of ventilation and hospitalization as well as with necrotizing enterocolitis. Establishment of protocols for the prevention of nosocomial infections during hospitalization in the NICUs and mechanical ventilation of preterm neonates is recommended.

**Supplementary Information:**

The online version contains supplementary material available at 10.1186/s12887-023-04359-6.

## Introduction

Neonatal sepsis is a clinical syndrome caused by pathogenic bacteria in the blood circulation and is characterized by hemodynamic changes and other systemic symptoms of infection. It may affect neonatal growth, result in organ failure and tissue damage, with implications for subsequent neurocognitive development and neonatal mortality [1]. The occurrence of neonatal bloodstream infection after the third day of life (> 72 h after birth) is defined as late-onset neonatal sepsis (LOS) and is a common complication in preterm neonates admitted to Neonatal Intensive Care Units (NICU) [2]. LOS is associated with poor prognosis accompanied by a prolonged hospital stay and significant morbidity and mortality, especially in preterm neonates with very low birth weight (VLBW) [3, 4].

Although incidence of LOS may vary among hospitalized neonates, increased survival of preterm and VLBW neonates has resulted in an overall increase of LOS incidence in recent years [5]. Invasive procedures employed in modern neonatal care are associated with higher risk of infection in this group of neonates [6], and previous studies highlighted placement of central venous catheters, prolonged mechanical ventilation, immunosuppression due to use of broad-spectrum antibiotics, necrotizing enterocolitis, patent ductus arteriosus and parenteral administration of nutrition as risk factors for LOS. [7–10]. Given the limited capacity of the developing immune system to mount an effective response to pathogens, which has been linked to increased neonatal susceptibility to infections [11], the mechanism of immunosuppression, caused by the use of broad-spectrum antibiotics, has been the focus of several studies. More specifically, early, and empirical antibiotic use has been found to result in detrimental effects on neonatal intestinal microbial diversity and richness, leading to intestinal dysbiosis [12, 13]. Based on a recent systematic review, these effects could range from acute to long-term, although additional evidence on the strength and duration of antibiotic-specific relationships with intestinal dysbiosis is needed [14]. Nevertheless, mounting evidence suggest that early-life acquisition of a dysbiotic intestinal microbiome predisposes to increased susceptibility to develop sepsis [15], emergence of resistance genes in the intestinal microbiome [16] and dysregulated immune responses [17].

A positive blood culture sets the diagnosis of LOS, and its incidence varies considerably across countries. In Australia, Canada, Greece, France and Finland, the incidence of LOS was estimated at 15.1%, 18.7%, 4.5%, 4.9% and 3.2%, respectively [18]. Although there are geographical differences in the distribution of pathogens isolated in blood cultures from preterm neonates with LOS in developed countries, gram-positive bacteria are the most common isolates with coagulase-negative staphylococci (CoNS) being the most common (> 30%) [5, 19]. Furthermore, the type of isolated pathogens is an independent prognostic factor for LOS in preterm neonates, with gram-negative bacteria demonstrating increased morbidity and mortality [20]. In an effort to better understand the epidemiology of neonatal infections, improve clinical practice and quality of care and establish better infection prevention strategies, several countries have set up national neonatal research networks for LOS [21–23].

Cyprus is an island with a population of around 1.216.000 people. The rate of caesarean section is 55.4% and of prematurity is 12%. Approximately, 65% of preterm neonates born in Cyprus require hospitalization in the NICU [24], but the frequency of LOS in the country remains unknown. To prevent or better manage of LOS in this resource limited setting, it is important to assess the frequency and complications of the condition as well as identify the prevailing prenatal, perinatal and postnatal risk factors for LOS in preterm infants. This approach may allow for earlier and more precise identification of LOS, leading to earlier and more targeted therapy with limited adverse events [25]. In this study, we examined the epidemiology of LOS in Cyprus, described the main clinical features and complications of neonates hospitalized with LOS in the country’s single neonatal tertiary care center and quantified the effect of possible risk factors for the development of LOS.

## Materials and methods

### Study population and study design

This was a prospective, case-control study conducted in the tertiary hospital ‘‘Archbishop Makarios III’’ [NAM III], which hosts the only level III NICU in Cyprus and cares for all preterm neonates in need of tertiary management in the country.

The study included all neonates with gestational age < 37 weeks, who were admitted in the NICU of NAM III between 1st April 2017 and 31st October 2018, and whose parents provided written informed consent to participate in the study. Participants were delivered either in the Obstetrics Department of NAM III, or in other public or private hospitals of the country and were then transferred to the NICU of NAM III for further management. Upon admission to the NICU, a blood culture was routinely performed in all neonates. If positive, a diagnosis of early neonatal infection (Early Onset Sepsis – EOS) was made and these were not included in the study population.

Based on the NAM III NICU clinical protocol, during hospitalization and upon clinical suspicion of bloodstream infection by the on-duty neonatologist, a blood culture was obtained, a lumbar puncture was performed (unless there are specific contraindications), and an empiric antibiotic treatment was initiated. Based on the results of blood cultures, preterm neonates were categorized into 2 separate groups, as in previous studies [4, 26–32]: (a) “Confirmed LOS” cases with positive blood culture - microorganism isolated at or after 72 h of life, LOS symptoms and who received blood culture-directed antibiotic therapy, (b) “Unconfirmed LOS” with clinical presentation of LOS and a negative blood culture taken at or after 72 h of life - no microorganism isolated. In the absence of international consensus on the definition of clinical LOS with negative blood culture in neonates [28, 29], we defined “Unconfirmed LOS” in the presence of all 4 of the subsequent criteria: [1] at least 2 of the following clinical signs and laboratory findings: rectal temperature > 38^0^ C or < 36^0^ C, leukocytosis or leukopenia, hypotension, bradycardia or tachycardia, or respiratory distress [shortness of breath or apnea], [2] absence of apparent infection at other site, [3] intake of antibiotics for at least 5 days and [4] no isolation of a microorganism in blood cultures [4, 30, 31]. A 3rd group of hospitalized preterm neonates served as “Controls” and included neonates without clinical symptoms suggestive of LOS and no confirmed infection in the blood culture routinely collected at time of NICU admission. Overall, we excluded from the study preterm neonates with early neonatal infection (positive blood culture during the first 72 h of life), congenital malformations, known chromosomal abnormalities, and infants with positive cultures of biological fluids other than peripheral blood. In addition, preterm neonates whose medical records had incomplete data of the above variables were also excluded from the study, as well as preterm neonates who died within the first 3 days of life. Lastly, preterm neonates with 2 microorganisms isolated in their blood culture, or if the microorganism was considered a microbiological contamination, were also excluded from the study [32].

### Data collection

The socio-demographic and medical data of the mothers and the clinical data of the participating preterm neonates from birth to discharge, were collected from the hospital medical records and recorded in an electronic database. Collected data included maternal age, nationality, body mass index, and smoking habits, obstetric complications (preeclampsia, eclampsia, gestational hypertension, premature rupture of membranes, vaginal bleeding during pregnancy, placental abnormalities, gestational diabetes mellitus-GDM, chorioamnionitis, maternal thyroid disease, intrauterine growth restriction-IUGR), characteristics of pregnancy and delivery (single or multiple pregnancy, regular obstetric monitoring, parity, place and mode of delivery, prenatal steroid administration, gestational age, neonatal birth weight), perinatal characteristics (Apgar score, resuscitation in the delivery room, bronchopulmonary dysplasia-BPD, necrotizing enterocolitis-NEC, retinopathy of prematurity-ROP, respiratory distress syndrome-RDS), invasive or therapeutic interventions and duration of hospitalization. According to NAM III NICU clinical protocol, premature infants are discharged if they weigh ≧ 2 kg, have no abnormal clinical manifestations and are adequately bottle- or breast-fed.

### Statistical analysis

Demographic and other characteristics of the mothers and newborns were presented as percentages in the case of categorical variables and medians (range) in the case of continuous variables. Comparisons between the 3 groups (LOS confirmed, LOS unconfirmed and Controls) were performed using Pearson’s Chi-squared test for categorical variables, Fisher’s exact test for continuous normally distributed variables and Kruskal-Wallis rank sum test for continuous non-normally distributed variables. The associations between demographic, clinical and treatment characteristics with LOS were assessed using univariate logistic regression. The dependent variable was binary and set as LOS positive status (LOS confirmed and LOS unconfirmed grouped together) or LOS negative status (controls). Multivariate logistic regression was performed including only the variables that were found to be statistically significant at p_value_ <0.1 at the previous step. All analyses were performed using the statistical software R version 4.0.3. [33]

## Results

364 preterm neonates were eligible for participation, and finally 350 were included in the study (96.2% participation rate). Among the 14 preterm neonates excluded, in 9 neonates the medical record was incomplete while in 5 parental consent was not obtained. A summary of the main maternal, perinatal, and postnatal characteristics of the study population is available in Table 1. Of the 350 neonates, 79 (22.6%) were classified as “confirmed LOS”, 65 (18.6%) as “unconfirmed LOS”, while the remaining 206 (58.8%) infants had no symptoms of LOS and were classified as “controls”. The frequency of “confirmed LOS” ranged from 10.9% in moderate to late preterm neonates (MLPT) to 78.6% in extremely pre-term (EPT) ones. Similarly, “confirmed LOS” was more frequent in preterm neonates of extremely low birthweight (ELBW) (70.6%), compared to preterm neonates with normal birthweight (9.4%). The most commonly isolated microorganism in the “confirmed LOS” group was CoNS (n = 72, 91.1%). The other microorganisms isolated were Candida species (n = 2), Enterococcus Faecalis (n = 2), Kocuria (n = 1), Sphingomonas paucimobilis (n = 1) and Escherichia coli (n = 1). There were no deaths in the participating neonates during hospitalization at the NICU.

**Table 1:** Participants characteristics (total sample, n = 350)

Maternal Characteristics	N (% of total)
Age (years)*	32 (28–35)
Cypriot nationality n (%)	280 (80%)
Body Mass Index, before pregnancy*	22.2 (19.9–25.6)
Smoking during pregnancy n (%)	33 (10%)
Regular prenatal monitoring, according to WHO guidelines	337 (97%)
Perinatal variables	
Delivery at tertiary hospital [Archbishop Makarios III]	176 (50%)
Vaginal delivery	33 (9%)
Multiple births	125 (36%)
Preeclampsia	63 (25%)
Vaginal bleeding	80 (24%)
Premature rupture of membranes	107 (31%)
Prolonged rupture of membranes (> 24 h)	48 (14%)
Gestational diabetes	55 (16%)
Steroids administration	238 (69%)
Postnatal variables	
Gestational age (weeks)*	33 (31–34)
Birth weight (gr)**	1895 (604)

*Median and Interquartile Range (IQR)

** Mean and Standard Deviation

### Maternal, pregnancy and neonatal characteristics of the 3 groups

The distribution of maternal and pregnancy characteristics in the 3 groups is presented in Table 2. Maternal age and frequency of IUGR differed between the 3 groups; “LOS confirmed” neonates were characterized by higher maternal age and higher frequency of IUGR. The distribution of other maternal factors such as smoking and regular antenatal visits, as well as the distribution of other pregnancy complications including gestational diabetes, preeclampsia and thyroid disease during pregnancy were not significantly different between the 3 groups. Main neonatal characteristics such as gender, multiple birth status and mode of delivery were not statistically different between the 3 groups. However, a significant association between LOS and gestational age was observed, with EPT and VPT neonates demonstrating a higher risk of LOS (both confirmed and unconfirmed) in comparison to moderate to late preterm infants. Similar trends were also observed for birthweight, Apgar score (1st minute) and requirement for resuscitation at birth (Table 3).

**Table 2:** Perinatal variables, comparisons between the different categories

Characteristic	Controls (n = 206)*	Unconfirmed LOS (n = 65)*	Confirmed LOS (n = 79)*	p-value**
**Cypriot nationality**	162 (79%)	53 (82%)	65 (82%)	0.75
**Maternal age [years]**				0.02
<=18	5 (2.4%)	2 (3.1%)	0 (0%)	
18–35	151 (74%)	54 (83%)	51 (65%)	
> 35	49 (24%)	9 (14%)	28 (35%)	
**Smoking during pregnancy**	21 (11%)	4 (6.5%)	8 (10%)	0.61
**Appropriate prenatal care**	198 (98%)	63 (97%)	76 (96%)	0.74
**Gestational diabetes**	34 (17%)	11 (17%)	10 (13%)	0.71
**Preeclampsia**	35 (17%)	9 (14%)	19 (24%)	0.24
**Chorioamnionitis**	10 (5.0%)	7 (11%)	8 (10%)	0.14
**Maternal thyroid disease**	27 (14%)	4 (6.2%)	11 (14%)	0.27
**IUGR**	68 (33%)	23 (37%)	38 (50%)	0.04
**Preterm premature rupture of membranes**	60 (29%)	23 (35%)	24 (30%)	0.63
**Preterm premature rupture of membranes (> 24 h before delivery)**	24 (12%)	12 (19%)	12 (16%)	0.35
**Steroid administration**	136 (67%)	42 (65%)	60 (76%)	0.28

*N (% of total)

**Pearson’s Chi-squared test; Fisher’s exact test

**Table 3:** Postnatal variable characteristics analysis

Characteristic	Controls (n = 206)*	Unconfirmed LOS (n = 65)*	Confirmed LOS (n = 79)*	p-value***
**Gender female**	105 (51%)	33 (51%)	47 (59%)	0.41
**Multiple births**	75 (36%)	20 (31%)	30 (38%)	0.63
**Vaginal delivery**	16 (7.8%)	10 (15%)	8 (10%)	0.19
**Delivery at ‘‘Archbishop Makarios III’’ Hospital**	92 (46%)	33 (53%)	51 (66%)	0.01
Gestational age (weeks)**	34.00 (33.00, 35.00)	32.00 (30.00, 33.00)	31.00 (29.00, 32.00)	< 0.001
**Gestational age (categorical)**				
Extremely preterm (< 28 weeks)	2 (1.0%)	1 (1.5%)	11 (14%)	
Very preterm (28 to < 32 weeks)	10 (4.9%)	29 (45%)	40 (51%)	
Moderate to late preterm (32 to < 37 weeks)	194 (94%)	35 (54%)	28 (35%)	
Birthweight (gr)**	2,108 (1,832, 2,414)	1,620 (1,310, 1,996)	1,310 (1,106, 1,669)	< 0.001
**Birthweight (gr, in categories)**				
Extremely low	1 (0.5%)	4 (6.2%)	12 (15%)	
Very low	17 (8.3%)	21 (32%)	39 (49%)	
Low	152 (74%)	32 (49%)	23 (29%)	
Normal	36 (17%)	8 (12%)	5 (6.3%)	
Apgar score at 1st minute**	8.00 (7.00, 9.00)	6.00 (5.00, 8.00)	6.00 (5.00, 8.00)	< 0.001
**Resuscitation at birth**	81 (39%)	51 (78%)	66 (84%)	< 0.001

*N (% of total)

**Median and Interquartile Range (IQR)

**Pearson’s Chi-squared test; Kruskal-Wallis rank sum test

### Treatment characteristics and complications of prematurity of the 3 groups

Treatment and hospitalization characteristics differed significantly between the 3 groups. More specifically, duration of hospitalization and duration of ventilation were longer in both LOS confirmed and LOS unconfirmed neonates compared to controls. Confirmed and unconfirmed LOS neonates were characterized by higher rates of transfusions, inotrope administration, as well as more frequent placement of umbilical and central line catheters. Complications of prematurity such as RDS, NEC, BPD and ROP were also more frequent in confirmed and unconfirmed LOS neonates compared to controls (Table 4).

**Table 4:** Hospitalization characteristics

Characteristic	Controls (n = 206)*	Unconfirmed LOS (n = 65)*	Confirmed LOS (n = 79)*	p-value***
**Mechanical ventilation (SIPPV)**	99 (48%)	56 (88%)	72 (90%)	< 0.001
Duration of ventilation (days)**	0.0 (0.0, 3.0)	4.0 (3.0, 7.0)	5.0 (3.0, 9.2)	< 0.001
Duration of hospitalization (days)**	15 (11, 21)	37 (23, 52)	53 (36, 70)	< 0.001
**Use of transfusions**	113 (59%)	55 (86%)	73 (91%)	< 0.001
**Use of inotropes**	57 (28%)	40 (62%)	53 (66%)	< 0.001
**Use of umbilical catheters**	99 (48%)	56 (88%)	73 (91%)	< 0.001
**Use of central line catheters**	16 (8.4%)	20 (32%)	37 (47%)	< 0.001
**Use of Hickman catheter**	1 (0.5%)	2 (3.2%)	7 (9.0%)	0.001
**Necrotizing enterocolitis**	12 (6.4%)	18 (29%)	25 (31%)	< 0.001
**Bronchopulmonary dysplasia [BPD]**	4 (2.0%)	14 (22%)	41 (51%)	< 0.001
**Intraventricular hemorrhage**	0 (0%)	1 (2.5%)	1 (1.6%)	0.24
**Retinopathy of prematurity**	2 (1.2%)	6 (10%)	15 (19%)	< 0.001

*N (% of total)

**Median and Interquartile Range

***Pearson’s Chi-squared test;

Kruskal-Wallis rank sum test; Fisher’s exact test

### Association of risk factors and complications with LOS

To evaluate the direction and magnitude of the association between the examined risk factors and assessed complications with LOS, we grouped confirmed and unconfirmed LOS infants together and carried out univariate and multivariate logistic regression. In the univariate analysis, statistically significant associations with LOS were observed for gestational age, IUGR, Apgar score, resuscitation at birth, RDS, ventilation duration, use of catheters, transfusions, inotrope administration and duration of hospitalization (Table 5). In addition, LOS was significantly associated with NEC, ROP and BPD. All variables that were found to be significantly associated with LOS in the univariate analysis, were included as predictor variables in the multivariate model. An independent statistically significant relationship persisted only for duration of hospitalization, duration of ventilation, and NEC (Table 6).

**Table 5:** Univariate logistic regression results LOS confirmed and unconfirmed LOS are merged in one category and compared vs. controls:

Characteristic	OR (95%CI)	p-value
Maternal age	1.01(0.97–1.05)	6.56 × 10^− 01^
Delivery at tertiary hospital (Archbishop Makarios III)	1.78(1.14–2.76)	1.00 × 10^− 02^
Caesarean delivery	0.59(0.29–1.2)	1.45 × 10^− 01^
Gestational age	0.53(0.46–0.61)	2.69 × 10^− 18^
Birthweight (baseline normal)		
Extermely low	44.31(5.33-368.21)	4.50 × 10^− 04^
Very low	9.77(4.25–22.46)	7.85 × 10^− 08^
Low	1(0.49–2.03)	9.95 × 10^− 01^
Multiple birth	0.93(0.6–1.45)	7.40 × 10^− 01^
Sex	0.83(0.54–1.28)	3.98 × 10^− 01^
BMI normal	0.8(0.51–1.24)	3.18 × 10^− 01^
Smoking during pregnancy	0.77(0.37–1.63)	4.97 × 10^− 01^
Preeclampsia	1.16(0.67–2.01)	6.00 × 10^− 01^
Vaginal bleeding	1.36(0.82–2.25)	2.40 × 10^− 01^
Premature rupture of membranes	1.18(0.74–1.87)	4.80 × 10^− 01^
Steroids administration	1.2(0.75–1.9)	4.48 × 10^− 01^
Gestation diabetes	0.85(0.47–1.54)	5.97 × 10^− 01^
Maternal thyroidoid gland disease	0.75(0.38–1.47)	4.10 × 10^− 01^
IUGR	1.55(1-2.42)	5.20 × 10^− 02^
Chorioamnionitis	2.21(0.96–5.08)	6.00 × 10^− 02^
Apgar score at 1 min	0.65(0.57–0.74)	4.24 × 10^− 10^
Apgar score at 5 min	0.63(0.5–0.79)	4.48 × 10^− 05^
Resuscitation at birth	6.69(4.04–11.06)	1.36 × 10^− 13^
Mechanical ventilation	8.65(4.81–15.56)	6.01 × 10^− 13^
Duration of ventilation [SIPPV]	1.54(1.39–1.71)	1.30 × 10^− 16^
Duration of hospitalization	1.1(1.07–1.12)	3.10 × 10^− 20^
Transfusion	5.59(3.09–10.13)	1.34 × 10^− 08^
Inotropes	4.7(2.97–7.44)	3.60 × 10^− 11^
Umbilical catheters	9.3(5.1-16.95)	3.41 × 10^− 13^
Peripherally inserted central venous catheters [PICC]	7.29(3.95–13.45)	2.00 × 10^− 11^
Duration of parenteral nutrition	1.21(1.16–1.27)	3.39 × 10^− 19^
Hickman line [central venus catheter]	12.82(1.6-102.38)	1.61 × 10^− 02^
Necrotizing enterocolitis	6.31(3.18–12.52)	1.39 × 10^− 07^
Bronchopulmonary dysplasia	30.9(10.86–87.88)	1.25 × 10^− 10^
Retinopathy of prematurity	14.57(3.35–63.35)	3.52 × 10^− 04^

**Table 6:** Multivariate logistic regression results for LOS (LOS confirmed and unconfirmed LOS are merged in one category) vs. controls:

Characteristic	OR (95%CI)	p-value
Gestational age	0.91 (0.68–1.22)	5.21 × 10^− 01^
IUGR	1.17 (0.52–2.61)	7.60 × 10^− 01^
Apgar score 1st minute	0.9 (0.73–1.12)	3.61 × 10^− 01^
Resuscitation at birth	1.28 (0.52–3.15)	5.86 × 10^− 01^
Duration of ventilation (SIPPV)	1.23 (1.07–1.43)	4.87 × 10^− 03^
Duration of hospitalization	1.06 (1.01–1.10)	1.02 × 10^− 02^
Transfusion	1.45 (0.58–3.60)	4.25 × 10^− 01^
Inotropes	1.14 (0.55–2.37)	7.26 × 10^− 01^
Necrotizing enterocolitis	3.41 (1.13–10.25)	2.93 × 10^− 02^
Retinopathy of prematurity	0.28 (0.04–2.14)	2.18 × 10^− 01^
Bronchopulmonary dysplasia	1.92 (0.30-12.31)	4.92 × 10^− 01^

## Discussion

In this study, we report the frequency of LOS in premature neonates from the only one level 3 NICU in Cyprus, which covers the entire population of the Republic of Cyprus. In this population, the incidence of LOS was 41%, which is slightly higher than previously published estimates in Europe (20–38%) [26]. Our findings are in agreement with previous reports that demonstrated an association of LOS incidence in the NICU with birth weight and gestational age, ranging from 70% in very low birth weight (VLBW: ≤1500 g) neonates to 10.9% in MLPT (GA: 32–37 weeks) ones [34].

LOS in preterm neonates has important health and financial implications. Following nosocomial infections the duration of NICU hospitalisation of preterm neonates is increased, even with successful treatment [35, 36], thus increasing the financial cost of the overall inpatient care [37–39]. Our study showed that longer duration of mechanical ventilation is associated with increased risk of LOS, which is in-line with findings from similar studies and studies on ventilator associated pneumonia (VAP), an infection affecting intubated neonates [40, 41]. Characteristically, a recent study demonstrated that in neonates with VAP, the duration of mechanical ventilation was significantly longer compared to neonates without VAP, while the VAP mortality rate was estimated at 9% [42]. Similarly, newborns with RDS in mechanical ventilation have increased mortality, which may also be associated with LOS [43]. Introduction and diligent implementation of practices that reduce the frequency of LOS in NICU, especially in intubated neonates, are expected to lead to better clinical outcomes [44].

Other factors that may affect the frequency of LOS in preterm neonates include pregnancy complications and neonatal characteristics. Lower gestational age and lower birth weight were associated with increased risk in the univariate analysis as reported in previous studies [27, 45] and a recent meta-analysis focusing on recourse limited settings [46]. Although the survival of preterm neonates has increased over time, the rates of complications remain significant, with nosocomial infections [47] constituting an important primary cause of death after the first week of NICU hospitalization [48, 49]. Falciglia et al., have recently shown that infants with BW < 750 g have increased rates of RDS, NEC, cerebral hemorrhage, and infections [50]. To reduce the incidence of these complications, practices for prevention of premature delivery-especially the non-iatrogenic ones- are important. These strategies should aim at improving the socio-economic factors that affect prematurity, such as better nutrition of the pregnant woman and minimization of maternal stress [51, 52].

Evidently, increased survival rates of preterm neonates, led to a higher percentage of them receiving blood transfusions and invasive medical procedures during NICU hospitalization [53]. Latest evidence from two large clinical trials, show that the percentage of pre-term neonates (< 1000 g) receiving at least one transfusion during neonatal admission may be as high as 90%. Nevertheless, even in populations with similar characteristics and transfusion guidelines, considerable variability in this percentage may exist [54]. In our study, we did not observe an independent association of transfusions with blood products (concentrated erythrocytes, fresh frozen plasma and platelets) with higher risk of LOS. However, several previous studies have demonstrated this association in preterm neonates [55, 56]. Nevertheless, given that the most common cause of frozen plasma transfusion is infection, and the most common indication for platelet transfusion due to thrombocytopenia is prematurity and infection, [57, 58], transfusions with blood products will remain a frequent and sometimes necessary practice in pre-term neonates. However, there is strong evidence that this treatment method is not without complications [59–63] and a more restrictive transfusion strategy may limit unnecessary transfusions without increasing morbidity or mortality in this patient group [64–66]. Finally, this study showed a statistically significant independent association between LOS and NEC. NEC is a complication of multifactorial etiology including infections [67–69], and the association between LOS and NEC in preterm neonates is most likely attributed to the immaturity of their immune system and the resultant abnormal microbiota gut colonization [70, 71]. Given the strong relationship of both LOS and NEC with mortality and morbidity, more research efforts should be undertaken on the role of probiotics administration in NICU, a practice that is not currently implemented in Cyprus [72–74]. The potential cost effectiveness of this approach for preventing NEC is significant [75], although the choice of optimal probiotic strain and appropriate feeding type remains unclear [76].

Overall, our study benefited from prospective recruitment, prospective data collection and the high participation rate. In addition, as the NAM III hospital NICU is the only tertiary referral center in the country, with universal access of the population to its services, our study population is representative with no obvious limitations by socioeconomic or other criteria. Finally, as data collection relied on available data in patients’ files and as a result, recall and observed bias were avoided. Overall, these characteristics enhance the generalizability of our results. However, the study is characterized by some limitations, with the most important one being the high percentage of LOS attributed to CoNS. Although CoNS is a frequently identifiable LOS pathogen [77–79], it is also a common blood culture contaminant. For this reason, a laboratory–confirmed bloodstream infection for CoNS requires 2 or more blood cultures drawn on separate occasions according to CDC/NHSN guidelines [31], although this was not the practice in our NICU. Like in other settings, low blood volume and the need to start antibiotic therapy without delay, only rarely allow two blood cultures to be taken before treatment is initiated [79]. Although blood collection was carried out by competent staff using strict aseptic techniques, there is a possibility that some of the subjects were misclassified (i.e., included in the “Confirmed LOS” group, while these should have been classified as “Unconfirmed LOS”). Nevertheless, the descriptive comparisons between the two groups provide some evidence for important differences in certain perinatal, postnatal and hospitalization characteristics between the “Confirmed LOS” and “Unconfirmed LOS” subjects. In addition, the main findings of this study, on the associations between demographic, clinical and treatment characteristics with LOS, emerged from the comparison of the “Controls” group as the LOS negative status with the “Confirmed LOS” and “Unconfirmed LOS” grouped together as the LOS positive status. As a result, any misclassification between the two LOS categories did not influence the main study findings. An additional limitation is the employed cross-sectional study design, as it was able to assess only short-term clinical outcomes and couldn’t examine the association of LOS with long-term health outcomes. Furthermore, the study did not systematically collect data on non-sepsis infections such as ventilator associated pneumonia, that would allow us to define a concurrent focus of infection that may have progressed to LOS [7]. As a result, we couldn’t include the distribution of ventilator associated pneumonia or other infectious foci as an additional factor in the multivariate analysis. In addition, although participation rate was high and the risk for selection bias is limited, the overall low sample size did not allow for the evaluation of rare outcomes. Finally, our study was restricted to LOS cases among preterm neonates and for an observation period of 18 months. Further studies, employing appropriate methodology and spanning to all suspected LOS cases and for a longer observation period, are required to fully describe the epidemiology and microbiology of LOS in Cyprus.

## Conclusions

In summary, the present study highlights the epidemiology of LOS in preterm neonates in Cyprus, identifies possible risk factors and associated clinical characteristics, and complications. For the prevention of LOS, establishment of protocols for the prevention of nosocomial infections during hospitalization in the NICUs and mechanical ventilation of preterm neonates is recommended.

### Electronic supplementary material

Below is the link to the electronic supplementary material.

Supplementary Material 1

## Data Availability

All data generated or analysed during this study are included in this published article (Supplementary file 1).
